# Enhancing Athlete Resilience: Preliminary Validation of the Sports Mind Inventory and the Impact of Yoga of Immortals on Sports-Related Stress

**DOI:** 10.3390/bs15101385

**Published:** 2025-10-12

**Authors:** Ishan Shivanand, Naakesh Dewan, Himanshu Kathuria, Sadhna Verma

**Affiliations:** 1 Department of Yoga, University of Silicon Andhra, Milpitas, CA 95035, USA; ishan@uofsa.edu; 2 SYC Infinite, San Francisco, CA 94043, USA; 3 Behavioral Health, GuideWell-Florida Blue, Jacksonville, FL 32246, USA; drnick@dewan.pro; 4 Department of Pharmacy, National University of Singapore, Singapore 117543, Singapore; himanshukathuria01@u.nus.edu; 5 Nusmetics Pte Ltd., E-Centre@Redhill, 3791 Jalan Bukit Merah, Singapore 159471, Singapore; 6 The Cincinnati Veterans Administration Hospital, University of Cincinnati College of Medicine, 234 Goodman Street, Cincinnati, OH 45267, USA

**Keywords:** Yoga of Immortals (YOI), athletic performance, Dewan sport inventory, sports resilience

## Abstract

The mental and emotional health of an athlete is crucial for their performance and well-being. Sports-related stress can significantly impair their mental health. Further, there were minimal tools available to measure Sports resilience, specifically during COVID-19 restrictions or earlier. This study reports the preliminary validation of the Sports Mind Inventory (SMI) in athletes from different geographical areas (*n* = 66), with the majority of participants from Mauritius, and tests the SMI in elite athletes practicing the Yoga of Immortals (YOI). YOI is a unique combination of specific yogic postures, breathing exercises, sound therapy & meditation, which has demonstrated benefit in improving measures of mental health. The exploratory factor analysis of the 24-item SMI resulted in a six-factor inventory. The confirmatory factor analysis of these six-factor SMI showed goodness-of-fit index (0.935), and Cronbach’s alpha coefficient (α) of 0.949, showing good fit and reliability. The correlation between overall scale and individual factors showed diverse degree of positive correlations. This validated SMI was then tested to investigate whether YOI can enhance athletes’ resilience to sports-related stress. Participants were a diverse set of athletes based in Mauritius who routinely engage in a wide range of athletic activities. Participants were randomly assigned to receive four weeks of YOI or no intervention. Both groups completed the SMI questionnaire at baseline and again after four weeks. The YOI intervention significantly increased (*p* = 0.002) the total mean SMI scores, and underlying factors, i.e., Factor 1: Positive and Competitive sports mindset (*p* = 0.014), Factor 2: Social relatedness and adaptability (*p* = 0.008), Factor 3: Resilient mindset and self-confidence (*p* = 0.036), Factor 4: Sports Resilience and Emotional Responses (*p* = 0.001). This indicated improved sports resilience and psychological health. No improvement was observed in the control group. The correlation analysis in YOI group at week-4 showed positive correlation between overall scales and underlying construct. In conclusion, SMI showed acceptable fitness to measure sport resilience. This YOI intervention helped in improving sports-related stress and improved athletes’ resilience.

## 1. Introduction

Peak athletic performance depends on not only physical health but also mental and emotional health. The stress of athletic training, whether at the professional or amateur level, can be considerable, especially for female athletes (Beisecker et al., 2024; Clark et al., 2025; Norris et al., 2025; Patel et al., 2010). The COVID-19 pandemic has worsened this stress with cancelled events, loss of compensation, and a lack of access to trainers (Gupta & McCarthy, 2021; McManama O’Brien et al., 2021). Prolonged stress can lead to sleep disruptions and eating disorders, both of which can negatively impact an athlete’s performance and overall health (Fullagar et al., 2015; Petisco-Rodriguez et al., 2020; Simpson et al., 2017; Watson, 2017).

Athletes require resilience, the ability to withstand and recover quickly from stress, for successful athletic performance (Cai et al., 2025; Costa et al., 2023; Predoiu et al., 2025). Athlete resilience is a constantly evolving journey influenced by personal psychological traits and the dynamic relationships athletes cultivate with their environment (Bicalho & Noce, 2020). It goes beyond merely bouncing back; it’s about thriving in the face of challenges and drawing strength both from within and from the world around them. Athletes with high resilience can manage stress more effectively and recover more quickly from injuries (Codonhato et al., 2018). Highly resilient athletes exhibit better health-related behaviors, well-being, and performance (Chrétien et al., 2024a). Improving resilience may enhance the athletic performance and overall quality of life of female athletes in particular (McManama O’Brien et al., 2021). Several tools are available to measure resilience (Codonhato et al., 2018; Fletcher & Sarkar, 2013; McManama O’Brien et al., 2021), including Connor and Davidson’s survey for adults (Chandler et al., 2020; Miller & France, 2021), the Sports Climate Questionnaire for semi-professional athletes (Trigueros et al., 2019), Richardson’s resilience model (Diotaiuti et al., 2020) and measures for adolescent resilience (Cousijn et al., 2018). However, there are currently minimal tools available to measure sports resilience, sports mental performance, and general life skills (Bicalho et al., 2022). In 2021, Camila et al. developed and validated a “Resilience Scale for Sport” (Bicalho et al., 2021). This scale consists of 15 questionnaires, and the study involved 30 athletes. However, the scale is in the early stages as it has not yet been tested with specific interventions. Furthermore, it does not integrate resilience with other constructs such as coping, self-confidence, motivation, and mental toughness. In 2022, another report from the same group presented a systematic review of research from 2008–2019 on resilience in athletes. Their finding also reveals a lack of a sport-specific resilience scale (Bicalho et al., 2022). Recently, Chrétien, A. et al. developed a RESilience qualities In SporT (RESIST) scale, with preliminary validation, covering the following dimensions of resilience: adaptability, positive personality, self-confidence, motivation, perceived social support (Chrétien et al., 2024b).

Numerous studies suggest that incorporating yoga and mindfulness techniques into athletic training can enhance athletes’ performance (Buhlmayer et al., 2017; Kadian & Shukla, 2023; Saeed et al., 2019; Tang et al., 2015; Vaidya et al., 2021). For example, a meta-analysis examining mindfulness practices among precision sport athletes, such as shooters and dart throwers, revealed that mindfulness improves both mental skills and overall performance (Buhlmayer et al., 2017). Another study demonstrated that yoga can improve the physical fitness, speed, and accuracy of cricketers (Vaidya et al., 2021). Moreover, meditative practices have been found to significantly enhance focus and attention, which are crucial elements of athletic performance (Krishnamurthy, 2023; Tang et al., 2015). Improved concentration can enhance movement efficiency while inadequate attention can even predict injuries (Junge, 2000). Additionally, yogic and meditative practices have been shown to reduce stress in athletes, including social anxiety (Saeed et al., 2019), which impacts around 15% of elite athletes (Gulliver et al., 2015). Despite these findings, there is currently no study that measure the effects of yoga and mindfulness on sports specific resilience, mental performance, and general life skills (Bicalho et al., 2021, 2022).

YOI app-based interventions have been shown to reduce clinical depression and anxiety (Verma et al., 2021b), insomnia (R. Tunuguntla et al., 2021; Verma et al., 2021a), and urinary incontinence (H. S. G. R. Tunuguntla et al., 2021). YOI also showed significant improvements in anxiety, depression, and insomnia among healthcare workers, highlighting the effectiveness of mental health interventions for this essential workforce (Currie et al., 2022). YOI is a unique combination of mindful physical, mental, and breathing exercises that has roots in ancient yoga traditions. However, the potential benefits of YOI for athletes have not been fully explored. Therefore, this study aimed to evaluate the effectiveness of YOI specifically for athletes, with a focus on sports-related mental health and resilience. To do so, the study employed the Sports Mind Inventory (SMI), a newly developed tool that measures sports resilience and psychological health in athletes. The study found that athletes who completed YOI training had significantly higher SMI scores, particularly in areas related to resilience, confidence, and a positive mindset. These results suggest that YOI app-based training is a promising intervention for improving athletes’ resilience to stress.

## 2. Materials and Methods

### 2.1. Sports Mind Inventory

The Sports Mind Inventory (SMI) was developed by an experienced Physician and sports psychiatrist. The SMI questionnaires were meticulously crafted to evaluate multiple domains, including the resilience concept. The questions were designed in a manner that is relevant, readable, clear, and appropriate for athletes. Each element was carefully considered to ensure that the questionnaires effectively engage athletes and gather meaningful data. It consists of 24-item self-report survey encompassing multiple domains, including the resilience concept, produced by N. Dewan. The 24-item survey is designed to cover six domains, measuring ‘Sports Resilience’, ‘positive sports mindset’, ‘purpose in life’, ‘social relatedness’, ‘positive and resilient mindset’, and ‘Sports Self-Confidence’ (Table 1).

The first six (1 to 6) questions measure ‘Sports Resilience’. It focuses on both cognitive and emotional responses to adverse situations in sports, such as losing. The next nine (7 to 15) questions measure ‘positive sports mindset’. It addresses well-established attitudinal, cognitive, and mental skills related to sports performance. The next question-16 measures ‘purpose in life’. The next six questions (17 to 22) measure the ‘social relatedness’ (17 and 18) and ‘positive and resilient mindset’ (19 to 22). It captures psychological constructs like social relatedness, positive and flexible thinking style, self-reliance, and higher purpose, that correlate with resilience in day-to-day life. The last two questions (23 and 24) measure the ‘Sports Self-Confidence’. It aims to capture the self-perception of the athlete in terms of their sense of physical capacity and skill mastery.

Both positive and negative response styles are included in this section to mitigate response set bias. The instrument is available as a “coach’s version” or a “parents’ version” to determine agreement amongst respondents. Responses are recorded on 5-point Likert-type scale, where ‘1’ stands for ‘strongly disagree’, ‘2’ for ‘disagree’, ‘3’ for ‘neither’, ‘4’ for ‘agree’, and ‘5’ for ‘strongly agree’. SMI scoring is regular for most of the questions, excluding questions 1, 2, 5, and 6, which are reverse-scored.

### 2.2. Study Approval

The study was approved by the Institutional Review Board, University of Cincinnati, Cincinnati, Ohio, United States of America (IRB approval number, 2020-0494).

### 2.3. Participants for SMI Validation

The professional athletes were recruited in mid-October 2021, a period when COVID-19 was spreading and restrictions were in place worldwide. An email containing a weblink was sent to 80 prospective participants to complete the online consent form, demographics questionnaires, and the Sports Mind Inventory (SMI). Participants who agreed to the consent form and completed the SMI questionnaires were considered for the assessment. The response was received from 66 participants. The participants’ demographics are shown in Table 2.

The responses from these participants were processed in MS Excel and analyzed. Processed data were exported, and exploratory factor analysis (EFA) was applied using JASP (Version 0.95.2). The number of factors was identified based on the eigenvalues above 1, and analysis was done using Promax rotation with the maximum likelihood factoring method. Assumption tests were also applied, including KMO test, bartlett’s test, Mardia’s test. Following the data from EFA, confirmatory factor analysis was performed to get outputs including reliability, fit measures, Kaiser-Meyer-Olkin (KMO) test, Bartlett’s test of sphericity. The reliability testing of SMI overall scale and individual factors were assessed using internal consistency by Cronbach’s alpha coefficient (α) and Coefficient ω. The correlation matrix was also calculated.

### 2.4. Study Design and Participants

The demographic data from SMI validation showed that most participants were based in Mauritius. Therefore, the further study was conducted on professional athletes based in Mauritius. The study was conducted from mid-October 2021 to mid-November 2021, a period when COVID-19 was spreading and restrictions were in place worldwide. Out of 66 participants in SMI validation, 13 participants who residency was not from Mauritius were excluded from participation in the study. Participants who agreed on the consent form and completed the baseline questionnaires on time were considered for this study. A total of 47 participants were included in the study and randomly assigned into the control (*n* = 18) and YOI group (*n* = 29), and YOI app access was provided to each study participant in the YOI group. Later, seven participants were excluded from the analysis because either they did not complete the post-survey at all or they failed to complete it within the specified time frame. The participants’ demographics are shown in Table 3. In this study, a variant of the YOI program was specifically designed for athletes to provide clear instruction to participants via live streaming. The session was conducted using a private weblink. Every day, all test group participants attended these guided YOI protocols, which had 30–45 min duration.

### 2.5. YOI Intervention

The app-based YOI program has been described previously (Currie et al., 2022; H. S. G. R. Tunuguntla et al., 2021; R. Tunuguntla et al., 2021; Verma et al., 2021a, 2021b). The central components consist of breathworks, whole body movements, postures, and mind training; however, they are done in combinations concurrently. In this study, a variant of the YOI program was specifically designed for athletes to provide clear instruction to participants via live streaming. Briefly, the protocol starts with the whole-body movements, followed by intense multiple types of breathwork in sequential manner, and last components are primarily relaxation and mind training. The protocol sequence is repeated multiple times in each session for at least three times. The physical components are designed to provide a massage to the body’s meridians, exercise areas that are not regularly exercised, increase blood flow, and activate lymphatic circulation. While the breathworks are designed to expand the lung capacity, and improve blood oxygenation. These physical and breathwork component prepares the participants for mind body training. Makes one more attentive and receptive. The session was conducted using a private weblink. The primary variation was the increased intensity of (YOI) components. This approach was driven by the understanding that the participants were elite athletes, whose exceptional skills and capabilities warranted a more rigorous and demanding practice.

### 2.6. Statistical Analysis

The data was analyzed with Microsoft Excel and GraphPad Prism 8. The descriptive statistics are shown as mean (±standard error of mean) and the percentage difference (**δ**. Wilcoxon matched-pairs signed-rank test was used for both control and YOI groups. Considering the positive effect of YOI in general and specific populations is known, it was expected to get either positive outcomes or no change in resilience; therefore, a one-tailed test was applied to the YOI group and control. Percentage changes obtained from matched participants were analysed using an unpaired Mann–Whitney test with 95% confidence interval and one-tailed calculation.

## 3. Results

### 3.1. SMI Demographics

The population for reliability testing encompassed athletes involved in the following activities: badminton, boxing, Brazilian ju jitsu, equestrian sports, football, golf, judo, karate, kun khmer, muay thai, running race, horseback riding, trekking, dancing, gymnastics, cricket, soccer, tennis, table tennis, Strength Training, and swimming. Some of the participants also reported more than two sports. Most participants were from Mauritius (71.2% *n* = 47), followed by India (18.18%, *n* = 12), and other countries (8.9% *n* = 7), with mixed ethnicity and ages (Table 1). 62.12% (*n* = 41) of participants were male and 37.87% (*n* = 25) were female. 43.93% (*n* = 29) of participants were in the age range of 18 to 25 years, 25.75% (*n* = 17) in the range of 26–36 years, and 15.15% (*n* = 10) in the range of 37–47 years.

### 3.2. Exploratory Factorial Analysis

The SMI consisted of 24 questionnaires that covered several aspects of athletes’ mindset, resilience, confidence, and purpose. The Kaiser-Meyer-Olkin (KMO) Test measures the adequacy of sample data for factor analysis (Supplementary Information S1). The overall Measure of Sampling Adequacy (MSA) was 0.816, indicating the sample is adequate for performing factor analysis. A KMO value above 0.7 is generally considered acceptable, while values closer to 1 suggest that the data is highly suitable for such analysis. Most of the questions had MSA values above 0.7, with several, like Q4 (0.889), Q8 (0.861), and Q15 (0.871), indicating robust sample adequacy. Conversely, Q12 (0.936) and Q10 (0.895) show high KMO values, indicating strong sampling adequacy. Questions Q1 and Q3 have lower MSA values of 0.693 and 0.423, respectively. Overall, these questions are likely to be reliable indicators of the SMI and can significantly contribute to the factor analysis. This implies that, while the overall sampling adequacy is good, some questions may need to be reevaluated with a larger sample size to assess their suitability or potential exclusion from factor analysis.

Bartlett’s Test of Sphericity assesses whether the correlation matrix of the dataset is significantly different from an identity matrix, indicating that the variables are unrelated. Bartlett’s Test Chi-squared (Χ^2^) from the statistics of 869.072 with 276 degrees of freedom and a *p*-value of < 0.001 indicates that the correlation matrix is not an identity matrix, suggesting that there are likely relationships among the variables and justifies the use of factor analysis for the dataset. Furthermore, Mardia’s Test of Multivariate Normality is also presented, which presents the key statistics for skewness and kurtosis.

The normal Chi-Squared test indicated a Χ^2^ value of 140.671 with 147 degrees of freedom. This test assesses the goodness of fit of the model to the data, and in the context of EFA, it helps to determine whether the observed correlations among variables significantly differ from what would be expected by the model being tested. Usually, a *p*-value < 0.05 in the Chi-Squared test implies that the model does not fit the data well, suggesting that the factors extracted may not adequately capture the underlying structure of the data, while a higher *p*-value indicates a good fit, and the model appropriately represents the correlations among the observed variables. However, other indices of model fit, such as the Comparative Fit Index (CFI), Root Mean Square Error of Approximation (RMSEA), and others, are also provided for more comprehensive understanding of how well the model explains the data. The RMSEA value of zero indicates an excellent fit, as RMSEA < 0.05 is considered indicative of a good fit, while values up to 0.08 can still be acceptable. Additionally, the Standardized Root Mean Square Residual (SRMR) is 0.041, which is below the commonly accepted threshold of 0.08 for indicating a good fit. The Tucker–Lewis Index (TLI) is 1.022. Typically, TLI values above 0.95 are considered indicative of a good fit, suggesting a strong model. The Comparative Fit Index (CFI) value is 1, also indicating a good fit. The Bayesian Information Criterion (BIC) of −475.209 also suggested a strong model, with lower or negative values indicating a better fit. This suggested that the model not only fits the data well but also avoids overfitting by maintaining parsimony.

### 3.3. Confirmatory Factorial Analysis

The results from the confirmatory factor analysis (CFA) provided insights into the model’s fit to the data. Most indexes improved after factor analysis compared to the unidimensional items inventory (Table 4). The CFA Chi-square statistic for the baseline model was 1005.751 with 276 degrees of freedom (Table 4, Supplementary Information S2). For the factor model, this fit was significantly improved, yielding a Chi-square of 353.666 with 237 degrees of freedom and a *p*-value of < 0.001. This significance indicates that the factor model provides a significantly better fit than the baseline model, accounting for the variance in the data more effectively. The additional fit measures indicate that the model exhibits a Comparative Fit Index (CFI) of 0.840, suggesting an acceptable fit. The Tucker–Lewis Index (TLI) and the Bentler-Bonett Non-Normed Fit Index (NNFI) both had values of 0.814, indicating a reasonable fit. The Bentler-Bonett Normed Fit Index (NFI) was at 0.648, suggesting a less optimal fit than other indices. The Parsimony Normed Fit Index (PNFI) at 0.557 and Bollen’s Relative Fit Index (RFI) of 0.590 also reflect moderate fit. The Bollen’s Incremental Fit Index (IFI) of 0.848 indicates a relatively good fit compared to others. The Relative Non-centrality Index (RNI) was the same as CFI at 0.840, supporting its adequacy. Other fit metrics, such as the RMSEA, show a value of 0.08 with a 90% confidence interval of (0.067, 0.105). This suggested that the model has an acceptable fit, as an RMSEA value below 0.06 is often considered a good fit, while values up to 0.08 are deemed acceptable. The associated *p*-value for RMSEA was 0.002, indicating that the null hypothesis of a close fit can be rejected.

Lastly, the SRMR was 0.083, indicating that the residuals are reasonably small, which further corroborates the model’s quality. Hoelter’s critical N indicates the minimum sample size for the model to maintain good fit under different significance levels, with figures of 51.342 for α = 0.05 and 54.403 for α = 0.01. Overall, the CFA suggests that the model provides valuable insights into the data structure, and the fit indices indicate that the model is acceptable but can be further optimized with a larger sample size to achieve an excellent fit.

The EFA yielded six factors, which were confirmed by CFA. Factor 1 included Q8, Q10, Q11, Q12, Q14, and Q15 (Table 5). The estimate for Q8 was 1.000, serving as the reference indicator. The loadings for other indicators are all statistically significant (*p* < 0.001), with Q12 having the highest loading (1.088), indicating a strong relationship with this factor. The confidence intervals for the estimates are narrow, indicating reliable estimates, with Q14 being slightly lower (0.693) but still significant. Overall, these results suggest that these items are closely related and effectively measure a common underlying construct. Factor 2 includes Q17, Q18, Q19, Q20, Q22, and Q23, where Q17 serves as the reference with a fixed loading of 1.000. All other indicators show statistically significant loadings, particularly Q19, which has the highest loading of 1.264, suggesting it is a strong indicator of this factor. The lower and upper bounds of the confidence intervals for all items indicate that the loadings are reliably estimated, further validating the relevance of these indicators in representing Factor 2. Factor 3 includes Q9, Q16, Q21, and Q24, where Q9 is the reference indicator with a loading of 1.000. The loadings for Q16 (0.998) and Q21 (0.959) are also significant (*p* < 0.001), indicating their strong relationship with Factor 3. The significant loadings suggest that these indicators effectively measure the factor, although Q24 shows a slightly lower loading (0.733), yet it remains statistically significant. Factor 4 includes Q1, Q2, Q5, and Q6, where Q1 (1.000) serves as the reference indicator. Q2 has a loading of 1.680, and Q5 has a loading of 1.820, both of which are significant, indicating that they effectively represent this factor. The statistical significance (*p* < 0.01) and substantial loadings suggest that these questions effectively measure this latent construct. Factor 5 includes Q4 and Q13, where Q4 (1.000) serves as a reference for the factor’s scaling. The Q13 showed a significant loading of 0.854, with a standard error of 0.157, resulting in a z-value of 5.442. This high z-value indicates that Q13 is a strong predictor of Factor 5, with a *p*-value < 0.001 indicating a statistically significant relation. Factor 6 includes Q3 and Q7, where Q3 (1.000) serves as the reference indicator. The Q7 showed a significant loading of 1.505, with a standard error of 0.795, resulting in a z-value of 1.893, and a *p*-value closer to 0.05, indicating a strong relationship between Q3 and Q7, and Q7 has a substantial influence. However, the relatively high standard error (0.795) might indicate some variability in the data. Overall, these results indicate that all factors and their corresponding indicators are statistically significant, providing robust evidence for the measurement model. The narrow confidence intervals indicate that the parameter estimates are reliable. Each factor represents a distinct construct, with the loading estimates affirming the strength and relevance of respective indicators. This analysis supports the validity of the model. The relatively high standard error in Q7 suggests considering a larger sample size for further optimization and an excellent fit.

### 3.4. Reliability Testing

The reliability testing for the individual modules and the overall scale, as analyzed through Cronbach’s alpha coefficients (α) and coefficient ω, is shown in Table 6. These coefficients are essential in assessing the internal consistency of the factors measured by the scale, ensuring that the items within each factor are reliably reflecting the underlying constructs. The overall scale showed α of 0.917 and ω of 0.949, revealing excellent consistency. The α value for the individual domain ranged from 0.453 to 0.884 (Table 6). These values indicate a strong internal consistency of the overall scale, as a Cronbach’s alpha greater than 0.9 suggests excellent reliability (Ahmad et al., 2024). The α values for individual modules were near 0.7 or higher, indicating acceptable to excellent internal consistency across the domains. For Factor 1, both the coefficient ω (0.887) and the coefficient α (0.884) indicate a high level of reliability. Factor 2 also shows good reliability, with coefficients of 0.860 for ω and 0.848 for α. In contrast, Factor 3 presents a coefficient ω of 0.708 and α of 0.732, indicating acceptable reliability. Likewise, Factors 4 and 5 showed acceptable reliability, with Factor 4 exhibiting a coefficient ω of 0.702 and α of 0.703, while Factor 5 had ω of 0.738 and α of 0.734. Factor 6 showed a coefficient ω of 0.472 and α of 0.453, indicating lower reliability. However, the overall scale with factor 6 shows good reliability; therefore, we kept the factor 6 in the SMI. Overall, most factors exhibit strong reliability; however, the lower scores in Factor 6 need further optimization to ensure accurate capturing of the constructs. A larger sample size would be considered for future studies to further optimize and ensure excellent reliability for all factors.

### 3.5. Correlation Matrix for Factors and Overall Score

**Figure 1:**
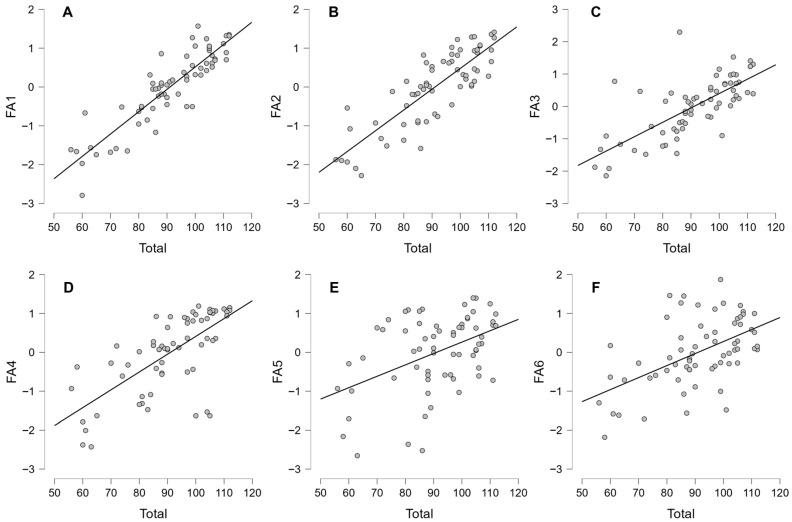
Correlation plot with data points (*n* = 66) of the total SMI score (1–24) with different domains. (**A**) Total vs. FA1, (**B**) Total vs. FA2, (**C**) Total vs. FA3, (**D**) Total vs. FA4, (**E**) Total vs. FA5, (**F**) Total vs FA6.

The correlations between the total score and Factors 1–6 (Table 7) demonstrated strong positive correlations with all the factors (FA1 to FA6) (Table 7 and Figure 1). This provides insight into the dataset’s dynamics, which can be further explored. The ‘r’ greater than 0.8 is considered a very strong correlation, and r in the range 0.6–0.8 is considered a strong correlation (Mocanu et al., 2024). Notably, FA1 showed the highest correlation with the total score, as indicated by a Pearson coefficient (r) of 0.891 and a Spearman coefficient (rho) of 0.883, both of which were significant at *p* < 0.001 (Table 7). This suggests that as scores in FA1 increase, the total score is likely to increase significantly as well. FA2 also showed a strong correlation (r = 0.834, rho = 0.82), reinforcing that this domain is also closely tied to the total score. FA5 presented a significant but lower correlation (r = 0.448, rho = 0.354), indicating that the relationship with the total score is weaker compared to FA1 and FA2. FA6 showed moderate correlations with the total score (r = 0.528, rho = 0.506), also significant at *p* < 0.001. Among the domain correlations, the strongest was observed between FA1 and FA2 (r = 0.691, rho = 0.685), indicating a significant and positive relationship between these two domains. FA3 exhibited a strong correlation with both FA2 (r = 0.615, rho = 0.636) and FA4 (r = 0.571, rho = 0.629), emphasizing that these domains are interconnected. The relations between FA4 and FA5 were weaker (r = 0.425, rho = 0.339). While FA3 and FA5 had a minimal correlation (r = 0.024, *p* = 0.846). Similarly, FA5 and FA6 demonstrated low correlations (r = 0.304, rho = 0.267). The confidence intervals for the correlation coefficients provide a range in which the true correlation is likely to lie, further confirming the reliability of these correlations. For instance, the confidence interval for FA1’s correlation with the total score (0.828 to 0.932) demonstrates robustness. In summary, FA1 and FA2 are key indicators associated with higher total scores, while revealing varying degrees of correlation among the other domains. This suggests that the scale encompasses a diverse set of non-overlapping measures to assess athletes’ resilience.

### 3.6. Effect of YOI on Athletes

The demographics show that most participants in SMI validation were based in Mauritius. Therefore, the YOI study was conducted only on professional athletes based in Mauritius. This enabled the logistics to be manageable because of the same time zones of participants, and facilitated better interaction between the YOI instructor and the participants. The YOI study population encompassed athletes involved in the following activities: badminton, boxing, Brazilian ju jitsu, equestrian sports, football, golf, judo, karate, kun khmer, muay thai, running race, horseback riding, trekking, dancing, gymnastics, and swimming. Some of the participants also reported more than two sports. All participants were from Mauritius with mixed ethnicity and ages (Table 3).

**Figure 2:**
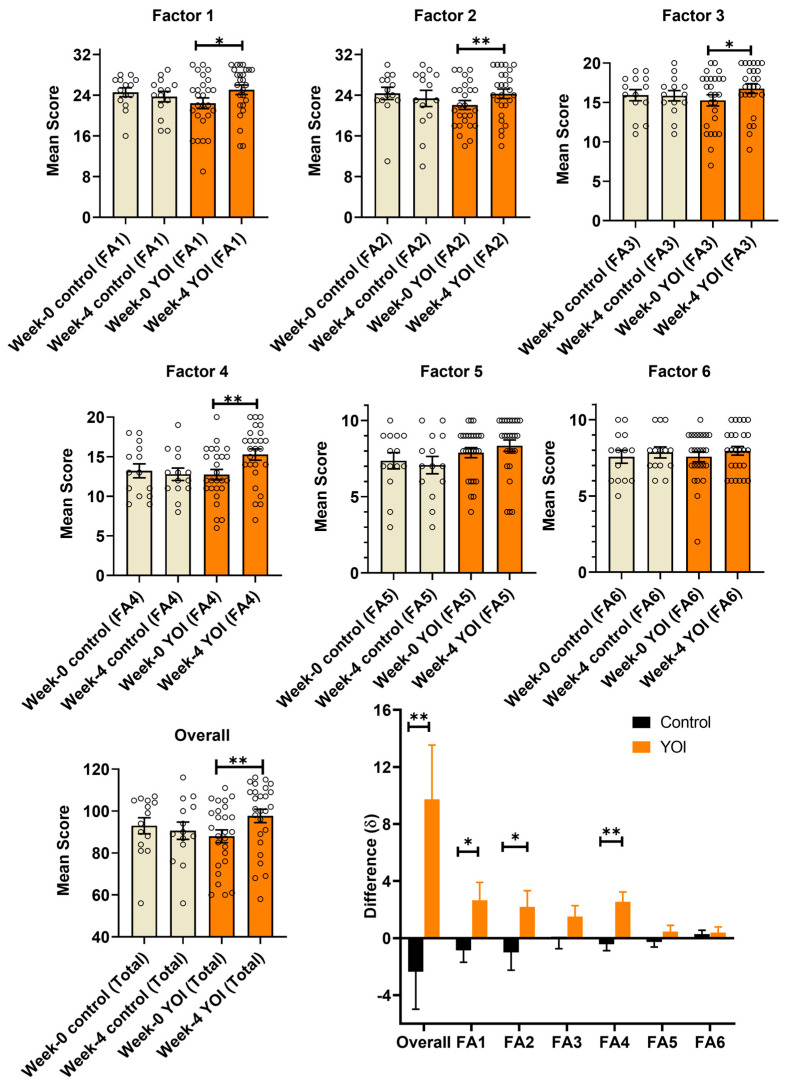
SMI score showing the effect of YOI on athlete sports-related mental health for the control group (*n* = 14) and the YOI group (*n* = 26) at week 0 and week 4. Wilcoxon matched-pairs signed-rank test was used to compare week 0 and week 4 SMI scores with 95% confidence interval. The bottom right shows difference (δ) in SMI score at week 4 from baseline among control and YOI group. Unpaired Mann–Whitney test compared control vs. YOI with 95% confidence interval. * *p* ≤ 0.05, ** *p* ≤ 0.011.

After four weeks of YOI, total SMI scores significantly increased in the YOI group (*p* = 0.002) while scores were non-significantly declined in the control group (Table 8 and Figure 2). The improved score after YOI demonstrates that this intervention improves resilience in athletes, and that the SMI can be used to measure this effect. In the YOI group, scores were significantly higher at week four compared to baseline for the following factors: Factor 1, Factor 2, Factor 3, and Factor 4 (Figure 2). The total mean difference in SMI scores was also significantly higher for YOI compared to the control group (*p* = 0.008). The mean differences were also significant for the sports resilience (*p* = 0.008), Factor 1 (*p* = 0.026), Factor 2 (*p* = 0.041), and Factor 4 (*p* = 0.002). The total mean difference in SMI scores was 14.15 ± 5.45% higher after YOI practices for four weeks, while a −2.24 ± 2.63% reduction was observed in the control group.

The correlation matrix for various factors (FA1 to FA6) and the total score within the YOI group at week-4 was calculated (Table 9). The Pearson and Spearman coefficients reveal a strong positive correlation across factors. The total score demonstrates exceptional correlations with the factors, particularly FA1, FA2, and FA3, all of which exhibit Pearson and Spearman coefficients above 0.95. This suggests that as the total SMI score in the YOI group increases, so do the scores in these factors, indicating a robust relationship among these measures. The confidence intervals (CIs) for these factors are narrow, ranging mostly from 0.927 to 0.990 for Pearson coefficients, which reinforces the reliability of these correlations. Further, the significance of these correlations, with *p*-values less than 0.001, shows that these relationships are statistically significant. FA4, FA5, and FA6 also showed significant correlations with the total score but exhibited somewhat lower coefficients (around 0.72 to 0.82 for FA6 and the others). The correlations among factors also reveal significant relationships. For instance, FA1 and FA2 have a Pearson coefficient of 0.924, indicating a high degree of correlation. Similarly, significant correlations are observed between FA2 and FA3 (0.955) and between FA4 and FA5 (0.838). Overall, these results are in agreement with the validation results, even though the sample size was small. Further, it confirms YOI effects on Athletes domains, i.e., Positive and Competitive sports mindset’, Social relatedness and adaptability, Resilient mindset and self-confidence, Sports Resilience and Emotional Responses, Positive Sports mindset and motivation, Enjoyment and Detachment from Outcome, are interconnected.

## 4. Discussion

Several other tools are available to measure resilience in general, including Connor and Davidson’s survey for adults (Chandler et al., 2020; Connor & Davidson, 2003; Miller & France, 2021), the Sports Climate Questionnaire for semi-professional athletes (Trigueros et al., 2019), Richardson’s resilience model (Diotaiuti et al., 2020), and measures for adolescent resilience (Cousijn et al., 2018). There are tools to measure sports performance as well (Adam et al., 2023; Jones et al., 2001). However, during the COVID-19 pandemic and earlier, there were limited tools available or specifically designed to measure sports resilience, sports mental performance, and general life skills. Self-assessment tools that have been used to identify mental health status of sports person include: Sports-Specific Resilience Scale (SSRS), Resilience Scale for Adults (RSA), Athletic Coping Skills Inventory (ACSI-28), Connor-Davidson Resilience Scale (CD-RISC), Athletic Resilience Questionnaire (ARQ), and Sports Resilience Questionnaire (SRQ). SSRS was first developed by Gucciardi, Gordon, and Dimmock in 2009 called Australian football Mental Toughness Inventory (Gucciardi et al., 2009). This scale consists of 24 items to assess sports resilience, such as perseverance, adaptability, and self-belief. Overall, the SSRS has been used as a reliable and valid tool for measuring resilience in athletes across various sports and contexts. However, SSRS has a potential issue, i.e., the scale may not be sensitive enough to detect changes in resilience over time (Blanco-Garcia et al., 2021; Den Hartigh et al., 2022). Another study used a Brief Resilience Scale (BRS) to examine the relationships between resilience, sport type, gender, age, and sport level among 1047 competitive athletes from five different sports, i.e., handball, basketball, volleyball, athletics, and judo (Blanco-Garcia et al., 2021). However, BRS is not specific to sports. Other assessment scales available before 2021 were not specific to athletes, except for the SMI presented in this study. In 2021, Camila et al. developed a “Resilience Scale for Sport” (Bicalho et al., 2021) consisting of fifteen questionnaires having five components: (1) Experiences, (2) Family Social Support, (3) Personal Resources and Competence, (4) Spirituality, and (5) Sport Social Support (Bicalho et al., 2021). In this study, for individual components, α was 0.64 to 0.77, and for total scores, α was 0.812, which shows acceptable consistency. However, the study mentions that the scale has not yet been tested with specific interventions. This scale does not include few components of resilience such as coping, self-confidence, motivation, and mental toughness. Another report from the same group reveals a lack of a sport-specific resilience scale (Bicalho et al., 2022) based on a systematic literature review. It is important to consider the athlete’s needs and goals, when selecting a resilience measurement tool. Though multiple tools can be used concurrently to gain a more comprehensive understanding, data processing can be challenging and troublesome.

Yoga and related modalities are ancient practices whose mental health benefits have been intuitively understood for millennia. Modern medical science has subsequently demonstrated multiple psychological and health benefits from such practices (Long Parma et al., 2015; Novaes et al., 2020). However, proper instruction, in a studio from a licensed teacher at a set time, is not feasible in all situations. Self-instruction is possible, but often minimally effective. YOI serves as a scalable instruction tool that can be accessed widely. YOI protocols in this study were explicitly tuned for athletes and directly taught by YOI founders and/or YOI teachers via live video streaming. Four weeks of YOI practice resulted in an increase in SMI scores (Table 8). The earlier research on YOI has shown that YOI reduces clinical symptoms of insomnia, depression, stress, and anxiety (Currie et al., 2022; R. Tunuguntla et al., 2021; Verma et al., 2021a, 2021b). Considering YOI combines whole body movements, postures, and mind training, the following could be the potential mechanism by which YOI shows these effects: increased oxygenation of the whole body and brain, increased oxygen intake capacity, improved blood circulation in the whole body, and lymphatic activation. These help improve detoxification throughout the entire body at the cellular level, including areas of the body that are not exercised regularly. This accelerated detoxification through the YOI protocol, along with high oxygenation, induces deep relaxation, which prepares the participants for mind–body training. Sangavi et al. have shown the effect of yogic exercise on cerebral hemodynamics, which can help improve the oxygenation of the brain (Sangavi et al., 2025). Moreover, cognitive function and performance are also directly linked to brain oxygenation (Williams et al., 2019). A recent study has shown that meditation increases oxygenation in the prefrontal cortex of the brain, which helps in better decision-making and impulse control (Ding et al., 2025). The studies have shown that mediation can significantly affect neurotransmitters (Krishnakumar et al., 2015; Thambyrajah et al., 2023). Particularly, it has been shown to improve the levels of serotonin and melatonin. Safdar et al. have mentioned the role of exercise-induced release of biomolecules from skeletal muscles and organs (Safdar et al., 2016). These biomolecules consist of peptides, nucleic acids referred to as exerkines (Li et al., 2024; Lou et al., 2022), which mediate the systemic adaptations. Furthermore, the sleep and glymphatic clearance have dynamic relationships, where better sleep quality has been linked to improved brain clearance via g-lymphatic clearance from nose (Reddy & van der Werf, 2020; Voumvourakis et al., 2023). Therefore, YOI effect on improved sleep can lead to better brain health and function, resulting in better sports resilience. The increased blood flow to brain can also help in better brain clearance because blood as a heat sink helps in clearance via g-lymphatic clearance pathway (Ferris, 2021). Additionally, mind body training helps in releasing stress, improves brain and nervous system, increases the capacity to handle uncertain situations, changes mental attitude and toughness, and improves clarity of thought.

## 5. Limitations/Future Research

The study has a few limitations that need to be considered. Firstly, it was conducted in a single country and had a small sample size of *n* = 66 for validation and *n* = 40 for intervention. However, it is difficult to recruit a large number of professional athletes for studies, especially when specific sports or geographic locations are involved. Furthermore, the study was conducted during a period when COVID-19 restrictions were in place worldwide. This also limited the recruitment of a large number of athletes from diverse geographical areas, specifically for the intervention. Therefore, in this study, the participants from different geographies were *n* = 66 only for validation, and the participants in the intervention were limited to participants from Mauritius only. This enabled us to manage logistics and synchronize participants’ time zones to deliver YOI protocols via live streaming, which also enhanced interaction between the YOI instructor and participants. However, for future studies involving larger groups, mobile or web applications could be employed to facilitate the study, enabling practice at a convenient time, as discussed in the existing literature. Often, reported studies involve less than one hundred participants, including control groups. Additionally, keeping all athletes involved in long-term studies is a significant challenge. One solution to this issue could be app-based interventions that have worldwide accessibility. However, to date, no such study has been conducted. Additionally, this study did not measure the impact of YOI on athletes’ sports performance. Therefore, it is unclear whether the observed improvements in SMI scores lead to enhancements in athletic performance.

This study presents the preliminary validation of SMI and shows the promising results of YOI in a specific geographical area and among a defined group of professional athletes. Future research will thoroughly validate the SMI using a larger number of athletes. To address the challenges faced in this study, future research will utilize mobile and web applications to reach various geographical locations and a broader range of professional athletes. Further studies can also evaluate the long-term effects of YOI on athletes’ resilience and mental health, providing valuable insights into its sustained efficacy over time. Such research would require a larger and more diverse participant pool that includes various sports disciplines, age groups, and skill levels. Additionally, it could explore the impact of YOI on concrete performance outcomes, such as injury rates, recovery times, and overall athletic performance metrics. The research may also examine the effects of YOI on specific populations, as different groups may respond differently to YOI, leading to tailored approaches that address their unique challenges.

## 6. Conclusions

This study represents the first evidence of the impact of YOI on the sports-related mental health of athletes. The results, as measured by the SMI score, demonstrate that YOI has a positive effect on the mental and emotional health of athletes. These findings suggest that YOI may be a valuable intervention for enhancing athletes’ resilience to sports-related stress. YOI emerges as a promising intervention for enhancing sports resilience.

## Figures and Tables

**Table 1:** List of Items in Sports Mind Inventory.

	If I am or my team is losing while I am competing in a sport, I tend to:
Q1	Blame myself
Q2	Look only at the negative side of things
Q3	Try not to think about it
Q4	Tell myself, I can come back no matter what
Q5	Get upset and emotional
Q6	Start giving up
	When I am competing in a sport:
Q7	I enjoy playing the sport even though I may not always win
Q8	I stay focused
Q9	I use breathing techniques to keep calm and focused
Q10	I tend to recover from my mistakes fast
Q11	I am smart about how to win
Q12	When I am done with a particular competition or event, I usually know what to work on to get better
Q13	I feel good/confident about myself regardless if I am winning or losing
Q14	I can get intense at the right moments
Q15	I fight hard to win and do the best I can
	In general, I believe that:
Q16	I have a purpose in life
Q17	I can talk to almost anyone
Q18	I fit in well with others
Q19	I am pretty flexible and can adapt to stressful situations
Q20	I have control over things in my life
Q21	I can figure things out when I have a problem
Q22	I can do things on my own if I have to
Q23	I am in excellent physical shape for my sport
Q24	I have strong fundamental skills in my sport

**Table 2:** Demographics of athletes (N = 66) included in SMI validation. It includes age, gender, ethnicity, and country, with participant number.

Category	N
Gender	
Female	25
Male	41
Age	
Above 18–25	29
26–36	17
37–47	10
48–58	6
59–69	4
Ethnicity	
American Indian or Alaska Native	1
Asian	29
Black or African American	5
Native Hawaiian or Other Pacific Islander	1
Other	19
White	11
Country	
Canada	1
France	1
India	12
Malaysia	1
Mauritius	47
Norway	1
South Africa	1
USA	2

**Table 3:** Demographics of athletes (n = 40) included in the YOI study. It includes age, gender, ethnicity, with participant number in each group.

Category	Control Group (N = 14)	YOI Group (N = 26)
**Gender**		
Female	8	13
**Male**	6	13
**Age**		
Above 18–25	5	10
26–36	2	7
37–47	2	6
48–58	3	1
59–69	2	2
**Ethnicity/Race**		
American Indian or Alaska Native	0	1
Asian	7	6
Black or African American	0	3
Native Hawaiian or Other Pacific Islander	0	1
Other	1	13
White	6	2

**Table 4:** List of various fit index values to assess goodness-of-fit and chi-square statistics for Undimensional (24 items) and six-factored (24 items distributed into Factor 1 to Factor 6).

Measures	Undimensional	Six-Factored
Comparative Fit Index (CFI)	0.723	0.840
Tucker–Lewis Index (TLI)	0.696	0.814
Bentler-Bonett Non-normed Fit Index (NNFI)	0.696	0.814
Bentler-Bonett Normed Fit Index (NFI)	0.551	0.648
Parsimony Normed Fit Index (PNFI)	0.503	0.557
Bollen’s Relative Fit Index (RFI)	0.508	0.590
Bollen’s Incremental Fit Index (IFI)	0.731	0.848
Relative Noncentrality Index (RNI)	0.723	0.840
Root mean square error of approximation (RMSEA)	0.112	0.087
RMSEA 90% CI lower bound	0.095	0.067
RMSEA 90% CI upper bound	0.128	0.105
RMSEA *p*-value	1.793 × 10^−8^	0.002
Standardized root mean square residual (SRMR)	0.096	0.083
Hoelter’s critical N (α = 0.05)	42.72	51.34
Hoelter’s critical N (α = 0.01)	45.19	54.40
Goodness of fit index (GFI)	0.638	0.935
McDonald fit index (MFI)	0.209	0.408
Expected cross validation index (ECVI)	8.406	8.118
Χ^2^ (Baseline Model)	1021.22	1005.75
df (Baseline model)	458.79	353.67
Χ^2^ (Factor Model)	276.00	276.00
df (Factor Model)	252.00	237.00
*p*-value	<0.001	<0.001

**Table 5:** Confirmatory factor analysis: Factor Loadings showing estimates, standard errors, z-values, and 95% confidence intervals (CI) for indicators across six factors.

Factor	Items	Estimate	Std. Error	z-Value	*p*	Lower CI	Upper CI
Factor 1: Positive and Competitive sports mindset’	Q8	1.000	0.000			1.000	1.000
	Q10	0.965	0.152	6.360	<0.001	0.668	1.263
	Q11	1.040	0.164	6.346	<0.001	0.719	1.362
	Q12	1.088	0.163	6.685	<0.001	0.769	1.407
	Q14	0.693	0.144	4.821	<0.001	0.411	0.974
	Q15	0.838	0.138	6.053	<0.001	0.567	1.109
Factor 2: Social relatedness and adaptability	Q17	1.000	0.000			1.000	1.000
	Q18	1.196	0.231	5.172	<0.001	0.743	1.649
	Q19	1.264	0.240	5.277	<0.001	0.794	1.733
	Q20	1.095	0.212	5.175	<0.001	0.680	1.510
	Q22	0.792	0.183	4.333	<0.001	0.434	1.150
	Q23	0.932	0.231	4.036	<0.001	0.480	1.385
Factor 3: Resilient mindset and self-confidence	Q9	1.000	0.000			1.000	1.000
	Q16	0.998	0.265	3.765	<0.001	0.478	1.517
	Q21	0.959	0.244	3.925	<0.001	0.480	1.438
	Q24	0.733	0.217	3.385	<0.001	0.309	1.157
Factor 4: Sports Resilience and Emotional Respons-es	Q1	1.000	0.000			1.000	1.000
	Q2	1.680	0.614	2.737	0.006	0.477	2.884
	Q5	1.820	0.690	2.637	0.008	0.468	3.173
	Q6	1.775	0.682	2.603	0.009	0.439	3.111
Factor 5: Positive Sports mindset and motivation	Q4	1.000	0.000			1.000	1.000
	Q13	0.854	0.157	5.442	<0.001	0.546	1.161
Factor 6: Enjoyment and Detachment from Outcome	Q3	1.000	0.000			1.000	1.000
	Q7	1.505	0.795	1.893	0.058	−0.053	3.064

**Table 6:** Cronbach’s alpha coefficient (α) for individual modules and the overall scale.

Factors	Domains	Coefficient ω	Coefficient α
Factor 1	Positive and Competitive sports mindset’	0.887	0.884
Factor 2	Social relatedness and adaptability	0.860	0.848
Factor 3	Resilient mindset and self-confidence	0.708	0.732
Factor 4	Sports Resilience and Emotional Responses	0.702	0.703
Factor 5	Positive Sports mindset and motivation	0.738	0.734
Factor 6	Enjoyment and Detachment from Outcome	0.472	0.453
Overall	SMI	0.949	0.917

**Table 7:** Correlation matrix of different domains with the total score (1–24) and other domains. ‘r’ is the Pearson coefficient, ‘rho’ is the Spearman coefficient, and CI is the confidence interval. N = 66.

Comparison			r		*p*-Value	Lower 95% CI	Upper 95% CI	rho		*p*-Value	Lower 95% CI	Upper 95% CI
Total	-	FA1	0.891	***	<0.001	0.828	0.932	0.883	***	<0.001	0.815	0.927
Total	-	FA2	0.834	***	<0.001	0.741	0.895	0.82	***	<0.001	0.722	0.886
Total	-	FA3	0.719	***	<0.001	0.578	0.819	0.729	***	<0.001	0.591	0.825
Total	-	FA4	0.689	***	<0.001	0.536	0.798	0.729	***	<0.001	0.592	0.826
Total	-	FA5	0.448	***	<0.001	0.231	0.623	0.354	**	0.004	0.122	0.549
Total	-	FA6	0.528	***	<0.001	0.328	0.683	0.506	***	<0.001	0.301	0.667
FA1	-	FA2	0.691	***	<0.001	0.539	0.799	0.685	***	<0.001	0.531	0.795
FA2	-	FA3	0.587	***	<0.001	0.402	0.726	0.633	***	<0.001	0.462	0.759
FA3	-	FA4	0.571	***	<0.001	0.381	0.714	0.629	***	<0.001	0.457	0.756
FA4	-	FA5	0.425	***	<0.001	0.204	0.605	0.339	**	0.006	0.105	0.537
FA5	-	FA6	0.325	**	0.008	0.09	0.526	0.32	**	0.009	0.084	0.521
FA2	-	FA3	0.615	***	<0.001	0.438	0.746	0.636	***	<0.001	0.466	0.761
FA3	-	FA4	0.498	***	<0.001	0.291	0.661	0.487	***	<0.001	0.278	0.652
FA4	-	FA5	0.142		0.256	−0.104	0.371	0.044		0.725	−0.2	0.283
FA5	-	FA6	0.304	*	0.013	0.067	0.509	0.267	*	0.03	0.027	0.478
FA3	-	FA4	0.311	*	0.011	0.075	0.514	0.334	**	0.006	0.1	0.533
FA3	-	FA5	0.024		0.846	−0.219	0.265	0.033		0.794	−0.211	0.273
FA4	-	FA6	0.194		0.118	−0.05	0.417	0.203		0.102	−0.041	0.424
FA4	-	FA5	0.385	**	0.001	0.158	0.574	0.379	**	0.002	0.151	0.569
FA5	-	FA6	0.417	***	<0.001	0.195	0.599	0.459	***	<0.001	0.244	0.631
FA5	-	FA6	0.498	***	<0.001	0.291	0.661	0.46	***	<0.001	0.246	0.632

* *p* < 0.05, ** *p* < 0.01, *** *p* < 0.001. FA1 to FA6: Factor 1 to Factor 6.

**Table 8:** Mean SMI scores, *p*-values, and mean difference from baseline for matched participants of the control group (N = 14) and YOI group (N = 26) at week-0 and week-4. Wilcoxon matched-pairs signed-rank test was used to compare week-0, and week-4 mean SMI scores using 95% confidence interval. Unpaired Mann–Whitney test compared control vs. YOI mean difference using 95% confidence interval.

	Mean SMI Scores						Mean Difference from Baseline		
	Week 0 (Control)	Week 4 (Control)	*p*-Value	Week 0 (Pre-YOI)	Week 4 (Post-YOI)	*p*-Value	δ-Control	δ-YOI	*p*-Value
Overall	93 ± 14.32	90.64 ± 15.52	0.291	87.96 ± 15.63	97.69 ± 16.3	0.002	−2.36 ± 9.82	9.73 ± 19.41	0.008
FA1	24.57 ± 3.3	23.71 ± 3.79	0.342	22.42 ± 5.39	25.08 ± 4.78	0.014	−0.86 ± 3.13	2.65 ± 6.37	0.026
FA2	24.36 ± 4.52	23.36 ± 5.94	0.278	22.08 ± 4.47	24.27 ± 4.72	0.008	−1 ± 4.69	2.19 ± 5.76	0.041
FA3	15.93 ± 2.65	15.86 ± 2.48	0.451	15.27 ± 3.6	16.77 ± 3.09	0.036	−0.07 ± 2.53	1.5 ± 3.94	0.214
FA4	13.21 ± 3.29	12.79 ± 2.94	0.241	12.73 ± 3.37	15.27 ± 3.64	0.001	−0.43 ± 1.7	2.54 ± 3.57	0.002
FA5	7.36 ± 1.99	7.07 ± 2.13	0.383	7.89 ± 1.68	8.35 ± 1.94	0.137	−0.29 ± 1.27	0.46 ± 2.21	0.171
FA6	7.57 ± 1.6	7.86 ± 1.35	0.172	7.58 ± 1.68	7.96 ± 1.46	0.305	0.29 ± 0.99	0.38 ± 2.04	0.734

**Table 9:** Correlation matrix of different domains with the total score (1–24) and factor 1 to factor 6 at week-4 in the YOI group. ‘r’ is the Pearson coefficient, ‘rho’ is the Spearman coefficient, and CI is the confidence interval. N = 24.

Comparison			r		*p*-Value	Lower 95% CI	Upper 95% CI	rho		*p*-Value	Lower 95% CI	Upper 95% CI
Total	-	FA1	0.965	***	<0.001	0.927	0.984	0.937	***	<0.001	0.869	0.970
Total	-	FA2	0.967	***	<0.001	0.930	0.984	0.943	***	<0.001	0.881	0.973
Total	-	FA3	0.978	***	<0.001	0.953	0.990	0.944	***	<0.001	0.883	0.974
Total	-	FA4	0.816	***	<0.001	0.642	0.910	0.638	***	<0.001	0.354	0.814
Total	-	FA5	0.816	***	<0.001	0.641	0.910	0.891	***	<0.001	0.779	0.948
Total	-	FA6	0.720	***	<0.001	0.481	0.860	0.619	***	<0.001	0.327	0.803
FA1	-	FA2	0.924	***	<0.001	0.843	0.964	0.862	***	<0.001	0.723	0.933
FA2	-	FA3	0.955	***	<0.001	0.906	0.979	0.909	***	<0.001	0.813	0.957
FA3	-	FA4	0.710	***	<0.001	0.465	0.854	0.476	**	0.009	0.133	0.717
FA4	-	FA5	0.838	***	<0.001	0.680	0.921	0.854	***	<0.001	0.710	0.930
FA5	-	FA6	0.674	***	<0.001	0.409	0.835	0.509	**	0.005	0.175	0.738
FA2	-	FA3	0.965	***	<0.001	0.926	0.984	0.922	***	<0.001	0.839	0.963
FA3	-	FA4	0.740	***	<0.001	0.513	0.871	0.538	**	0.003	0.214	0.756
FA4	-	FA5	0.810	***	<0.001	0.631	0.907	0.854	***	<0.001	0.710	0.930
FA5	-	FA6	0.690	***	<0.001	0.434	0.843	0.583	***	<0.001	0.275	0.782
FA3	-	FA4	0.747	***	<0.001	0.524	0.874	0.511	**	0.005	0.178	0.739
FA3	-	FA5	0.855	***	<0.001	0.711	0.930	0.899	***	<0.001	0.795	0.952
FA4	-	FA6	0.709	***	<0.001	0.463	0.854	0.587	***	<0.001	0.281	0.785
FA4	-	FA5	0.629	***	<0.001	0.341	0.809	0.471	**	0.01	0.126	0.714
FA5	-	FA6	0.484	**	0.008	0.142	0.722	0.390	*	0.036	0.028	0.662
FA5	-	FA6	0.671	***	<0.001	0.404	0.833	0.610	***	<0.001	0.313	0.798

* *p* < 0.05, ** *p* < 0.01, *** *p* < 0.001. FA1 to FA6: Factor 1 to Factor 6.

## Data Availability

The data presented in this study are available on request from the corresponding author. The data are not publicly available due to Ethical Concerns.

## Supplementary Materials

The following supporting information can be downloaded at: https://www.mdpi.com/article/10.3390/bs15101385/s1, S1. Additional results of Exploratory factor analysis, S2. Additional results of Confirmatory factor analysis.
