# Supplementary material for: Enhancing Athlete Resilience: Preliminary Validation of the Sports Mind Inventory and the Impact of Yoga of Immortals on Sports-Related Stress
Source: Behav Sci (Basel). 2025 Oct 12;15(10):1385. doi: 10.3390/bs15101385 (PMC12562012; doi:10.3390/bs15101385)
Supplement: Supplementary file 1 [file behavsci-15-01385-s001.zip › Supplementary Information_EFA and CFA.pdf]

Supplementary information

S1. Exploratory Factor Analysis

Kaiser-Meyer-Olkin Test

	MSA
Overall MSA	0.816
Q1	0.693
Q2	0.795
Q3	0.423
Q4	0.889
Q5	0.744
Q6	0.727
Q7	0.724
Q8	0.861
Q9	0.756
Q10	0.895
Q11	0.811
Q12	0.936
Q13	0.769
Q14	0.817
Q15	0.871
Q16	0.754
Q17	0.793
Q18	0.762
Q19	0.782
Q20	0.808
Q21	0.853
Q22	0.892
Q23	0.862
Q24	0.805

MSA: Measure of Sampling Adequacy

Bartlett's Test

X^2	df	p
869.072	276.000	< .001

Chi-Squared Test

	Value	df	p
Model	140.671	147	.631

Mardia's Test of Multivariate Normality

	Value	Statistic	df	p
Skewness	262.407	2,886.473	2,600	< .001
Small Sample Skewness	262.407	3,028.525	2,600	< .001
Kurtosis	627.870	0.445		0.656

Note. The statistic for skewness is assumed to be Chi^2 distributed and the statistic for kurtosis standard normal.

Factor Loadings

	**Factor 1**	**Factor 2**	**Factor 3**	**Factor 4**	**Factor 5**	**Factor 6**	**Uniqueness**
Q1	0.119	-0.099	-0.082	**0.209**	0.171	-0.026	0.879
Q2	0.042	0.236	0.027	**0.302**	0.226	0.110	0.542
Q3	-0.095	0.031	-0.060	-0.045	-0.071	**0.597**	0.704
Q4	0.292	0.098	0.190	0.145	**0.245**	0.153	0.393
Q5	0.200	0.104	-0.028	**0.318**	0.010	0.174	0.626
Q6	-0.333	0.007	0.084	**1.155**	0.017	-0.093	0.005
Q7	0.002	0.087	0.177	0.011	-0.157	**0.596**	0.596
Q8	**0.803**	0.034	-0.232	0.043	-0.059	0.335	0.235
Q9	-0.018	-0.323	**0.823**	0.028	0.222	0.311	0.316
Q10	**0.595**	-0.189	0.445	-0.058	0.137	0.160	0.253
Q11	**0.837**	0.122	-0.098	-0.224	0.209	-0.037	0.279
Q12	**0.818**	0.061	0.071	0.026	-0.169	-0.077	0.275
Q13	-0.149	0.195	0.357	0.003	**1.085**	-0.266	0.005
Q14	**0.738**	-0.041	0.236	-0.206	-0.113	-0.218	0.441
Q15	**0.643**	0.032	0.133	0.064	0.009	-0.171	0.434
Q16	0.112	0.124	**0.412**	0.299	-0.131	-0.100	0.497
Q17	0.097	**0.482**	-0.023	0.136	-0.164	0.147	0.584
Q18	0.176	**0.915**	-0.302	-0.112	0.162	-0.062	0.250
Q19	-0.294	**0.929**	0.046	0.051	0.070	0.214	0.192
Q20	0.122	**0.666**	-0.025	0.118	0.244	-0.208	0.321
Q21	0.057	0.273	**0.421**	0.359	-0.113	-0.190	0.316
Q22	0.271	**0.460**	0.216	-0.191	-0.130	-0.026	0.469
Q23	0.040	**0.395**	0.289	-0.218	0.117	0.192	0.585
Q24	0.002	0.031	**0.687**	-0.050	0.166	-0.071	0.524

Note. Applied rotation method is promax. Bold values are taken into factors 1-6.

Factor Characteristics

	Unrotated solution			Rotated solution		
	Eigenvalue	Proportion var.	Cumulative	SumSq. Loadings	Proportion var.	Cumulative
**Factor 1**	8.806	0.367	0.367	3.840	0.160	0.160
**Factor 2**	1.560	0.065	0.432	3.353	0.140	0.300
**Factor 3**	1.253	0.052	0.484	2.436	0.102	0.401
**Factor 4**	1.095	0.046	0.530	1.867	0.078	0.479
**Factor 5**	0.942	0.039	0.569	1.566	0.065	0.544
**Factor 6**	0.693	0.029	0.598	1.217	0.051	0.595

Factor Correlations

	Factor 1	Factor 2	Factor 3	Factor 4	Factor 5	Factor 6
**Factor 1**	1.000	0.640	0.517	0.567	0.411	0.271
**Factor 2**	0.640	1.000	0.569	0.472	0.108	0.246
**Factor 3**	0.517	0.569	1.000	0.272	-0.038	0.133
**Factor 4**	0.567	0.472	0.272	1.000	0.386	0.382
**Factor 5**	0.411	0.108	-0.038	0.386	1.000	0.492
**Factor 6**	0.271	0.246	0.133	0.382	0.492	1.000

Additional fit indices

RMSEA	RMSEA 90% confidence	SRMR	TLI	CFI	BIC
0.000	0 - 0.051	0.041	1.022	1.000	- 475.209

RMSEA: Root Mean Square Error of Approximation; SRMR: Standardized Root Mean Square Residual; TLI: Tucker-Lewis Index; CFI: Comparative Fit Index; BIC: Bayesian Information Criterion

Parallel Analysis

	Real data component eigenvalues	Simulated data mean eigenvalues
Q1*	9.159	2.266
Q2	1.987	2.054
Q3	1.570	1.872
Q4	1.521	1.715
Q5	1.247	1.596
Q6	1.054	1.460
Q7	0.964	1.355
Q8	0.873	1.259
Q9	0.736	1.175
Q10	0.604	1.075
Q11	0.595	1.009
Q12	0.532	0.944
Q13	0.494	0.874
Q14	0.414	0.807
Q15	0.370	0.739
Q16	0.311	0.670
Q17	0.289	0.600
Q18	0.273	0.531
Q19	0.250	0.474
Q20	0.200	0.419
Q21	0.196	0.355
Q22	0.157	0.310
Q23	0.118	0.250
Q24	0.083	0.191

Note. '*' = Factor should be retained. Results from PC-based parallel analysis.

Path Diagram

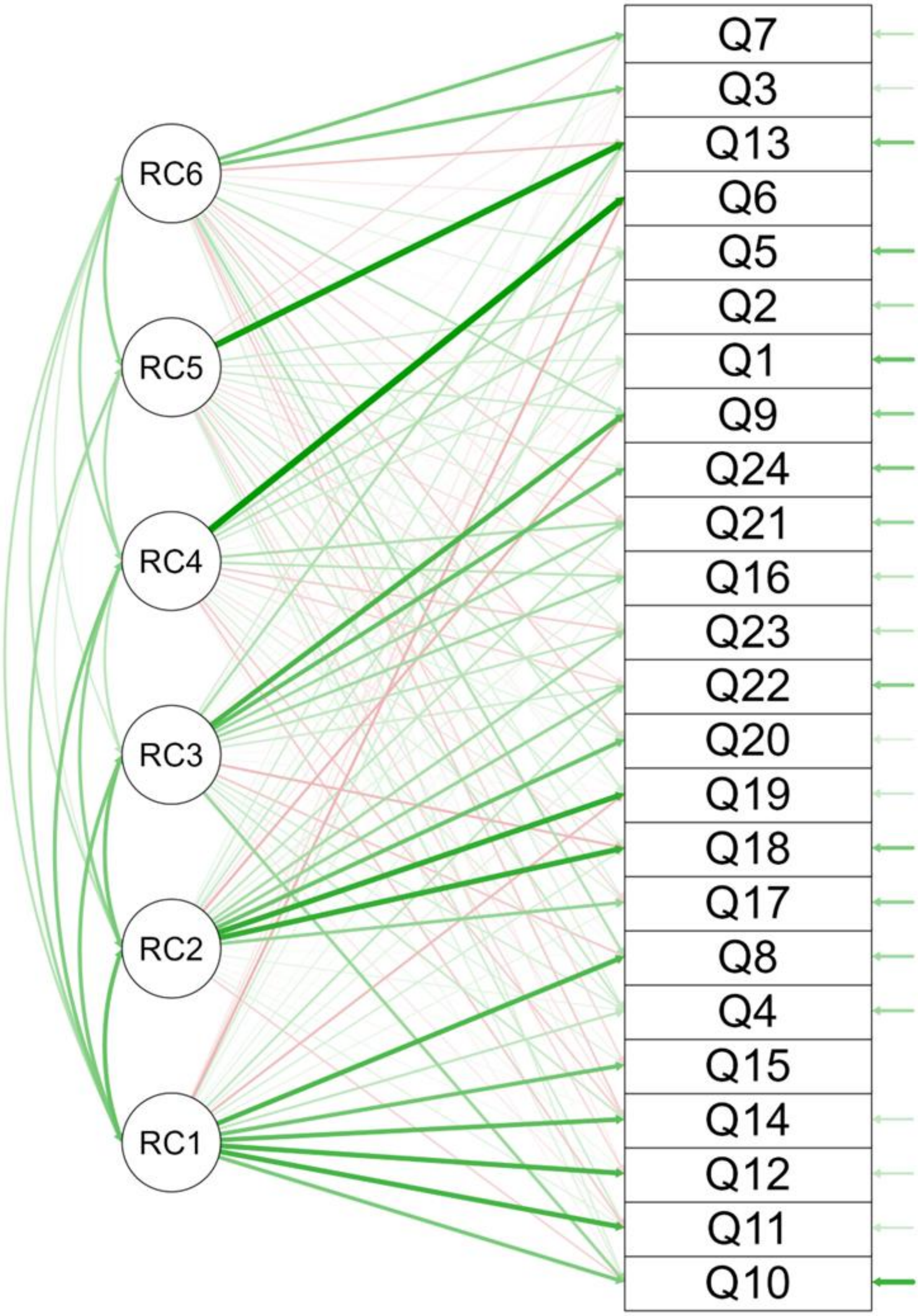

Scree plot

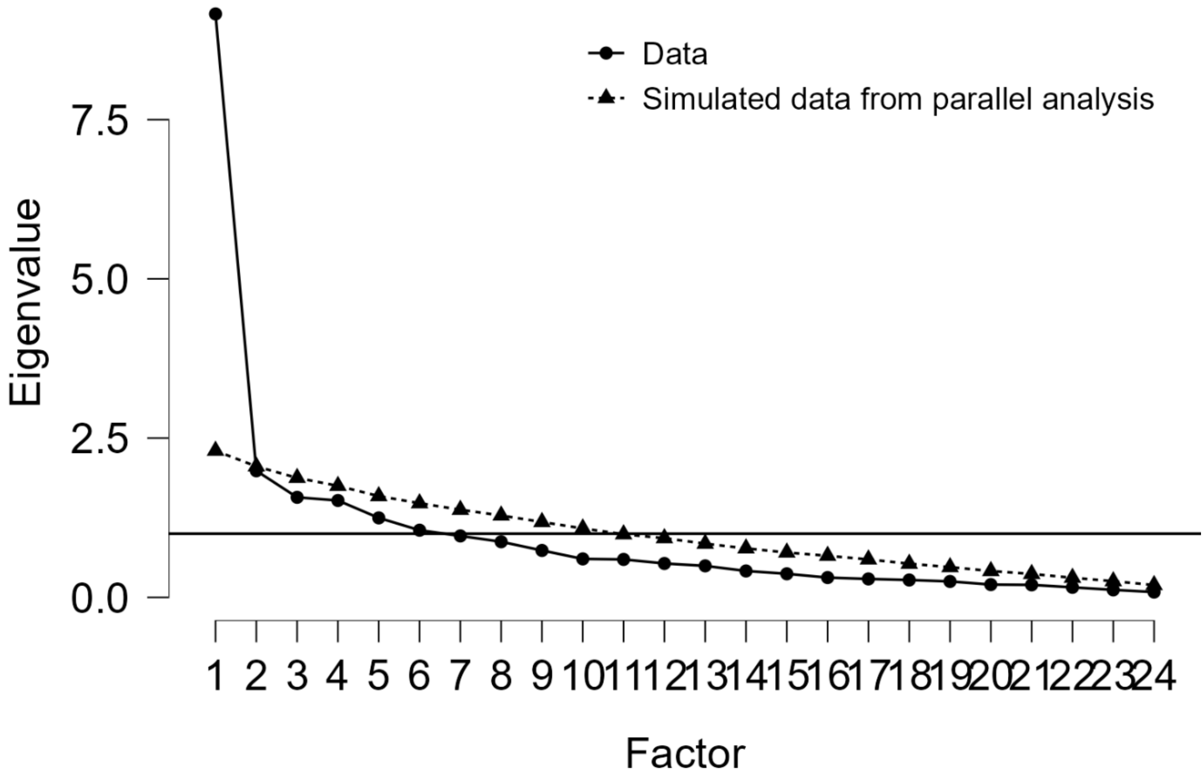

Data
Simulated data from parallel analysis
7.5
5.0
2.5
0.0
Eigenvalue
1 2 3 4 5 6 7 8 9 10 11 12 13 14 15 16 17 18 19 20 21 22 23 24
Factor

S2. Confirmatory Factor Analysis
Model fit

Chi-square test

Model	X^2	df	p
Baseline model	1,005.751	276	
Factor model	353.666	237	< .001

Note. The estimator is ML. The test statistic is standard. The standard error method is standard.

Additional fit measures

Fit indices

**Index**	**Value**
Comparative Fit Index (CFI)	0.840
Tucker-Lewis Index (TLI)	0.814
Bentler-Bonett Non-normed Fit Index (NNFI)	0.814
Bentler-Bonett Normed Fit Index (NFI)	0.648
Parsimony Normed Fit Index (PNFI)	0.557
Bollen's Relative Fit Index (RFI)	0.590
Bollen's Incremental Fit Index (IFI)	0.848
Relative Noncentrality Index (RNI)	0.840

Other fit measures

**Metric**	**Value**
Root mean square error of approximation (RMSEA)	0.087
RMSEA 90% CI lower bound	0.067
RMSEA 90% CI upper bound	0.105
RMSEA p-value	0.002
Standardized root mean square residual (SRMR)	0.083
Hoelter's critical N ($\alpha = .05$)	51.342
Hoelter's critical N ($\alpha = .01$)	54.403
Goodness of fit index (GFI)	0.935
McDonald fit index (MFI)	0.408
Expected cross validation index (ECVI)	8.118

R-Squared			
	R^2		R^2
Q1	0.138	Q13	0.443
Q2	0.651	Q14	0.367
Q3	0.175	Q15	0.561
Q4	0.768	Q16	0.519
Q5	0.452	Q17	0.402
Q6	0.413	Q18	0.616
Q7	0.495	Q19	0.652
Q8	0.557	Q20	0.617
Q9	0.265	Q21	0.636
Q10	0.614	Q22	0.391
Q11	0.611	Q23	0.330
Q12	0.673	Q24	0.349

Factor variances

					95% Confidence Interval	
**Factor**	**Estimate**	**Std. Error**	**z-value**	**p**	**Lower**	**Upper**
Factor 1	0.646	0.189	3.422	< .001	0.276	1.016
Factor 2	0.476	0.175	2.727	0.006	0.134	0.818
Factor 3	0.477	0.233	2.053	0.040	0.022	0.933
Factor 4	0.224	0.159	1.407	0.159	-0.088	0.536
Factor 5	0.744	0.190	3.922	< .001	0.372	1.117
Factor 6	0.232	0.177	1.308	0.191	-0.116	0.580

Reliability

**Factors**	**Coefficient ω**	**Coefficient α**
Factor 1	0.887	0.884
Factor 2	0.860	0.848
Factor 3	0.708	0.732
Factor 4	0.702	0.703
Factor 5	0.738	0.734
Factor 6	0.472	0.453
Total	0.949	0.917
