# Supplementary material for: Enhancing Athlete Resilience: Preliminary Validation of the Sports Mind Inventory and the Impact of Yoga of Immortals on Sports-Related Stress
Source: Behav Sci (Basel). 2025 Oct 12;15(10):1385. doi: 10.3390/bs15101385 (PMC12562012; doi:10.3390/bs15101385)
Supplement: Supplementary file 1 [file behavsci-15-01385-s001.zip › Supplimantary Info_Dewan Sports Mind Inventory.pdf]

The Sports Mind Inventory
By
N.A. Dewan, M.D.

Number ________________________

Age____________________________

Gender_________________________

Primary Sport __________________

If I am or my team is losing while I am competing in a sport, I tend to:

	Strongly Disagree	Disagree	Neither	Agree	Strongly Agree
1. Blame myself	1	2	3	4	5
2. Look only at the negative side of things	1	2	3	4	5
3. Try not to think about it	1	2	3	4	5
4. Tell myself, I can comeback no matter what	1	2	3	4	5
5. Get upset and emotional	1	2	3	4	5
6. Start giving up	1	2	3	4	5

When I am competing in a sport:

	Strongly Disagree	Disagree	Neither	Agree	Strongly Agree
7. I enjoy playing the sport even though I may not always win	1	2	3	4	5
8. I stay focused	1	2	3	4	5
9. I use breathing techniques to keep calm and focused	1	2	3	4	5
10. I tend to recover from my mistakes fast	1	2	3	4	5
11. I am smart about how to win	1	2	3	4	5
12. When I am done with a particular competition or event, I usually know what to work on to get better	1	2	3	4	5

13. I feel good/confident about myself regardless if I am winning or losing	1	2	3	4	5
14. I can get intense at the right moments	1	2	3	4	5
15. I fight hard to win and do the best I can	1	2	3	4	5

The Sports Mind Inventory
(Continued)

In general, I believe that:

	**Strongly Disagree**	**Disagree**	**Neither**	**Agree**	**Strongly Agree**
16. I have a purpose in life	1	2	3	4	5
17. I can talk to almost anyone	1	2	3	4	5
18. I fit in well with others	1	2	3	4	5
19. I am pretty flexible and can adapt to stressful situations	1	2	3	4	5
20. I have control over things in my life	1	2	3	4	5
21. I can figure things out when I have a problem	1	2	3	4	5
22. I can do things on my own if I have to	1	2	3	4	5
23. I am in excellent physical shape for my sport	1	2	3	4	5
24. I have strong fundamental skills in my sport	1	2	3	4	5

Copyright N.A. Dewan, M.D. 2014
